# Symptom network analysis in maintenance hemodialysis patients: a multicenter study

**DOI:** 10.7717/peerj.21741

**Published:** 2026-09-23

**Authors:** Xiaorong Liu, Huifen Zhao, Ziqing Hong, Yumei Peng, Jianqing Zheng, Suzhen Xie, Shuifeng Chen

**Affiliations:** 1 Department of Hemodialysis, Second Affiliated Hospital of Fujian Medical University, Quanzhou, China; 2 Clinical Nursing Teaching and Research Department, Second Affiliated Hospital of Fujian Medical University, Quanzhou, China; 3 Fujian Medical University, Fuzhou, Fujian, China; 4 The First Affiliated Hospital of Xiamen University, Xiamen, China; 5 Department of Radiation Oncology, Second Affiliated Hospital of Fujian Medical University, Quanzhou, China

**Keywords:** Maintenance hemodialysis, Symptom group, Symptom network, Study

## Abstract

**Objective:**

To identify symptom clusters and construct a symptom network in multicenter maintenance hemodialysis (MHD) patients, with the aim of elucidating core symptoms and informing evidence-based symptom management strategies.

**Methods:**

A cross-sectional study was conducted using convenience sampling to recruit 502 MHD patients from 10 hemodialysis centers in Quanzhou, Fujian Province, China. Symptom burden was assessed using the Dialysis Symptom Index. Exploratory factor analysis was employed to identify symptom clusters. Network analysis was performed using R software (version 4.3.1), with the qgraph, bootnet, and network tools packages utilized for network visualization, stability analysis, and centrality estimation. Network centrality indices were calculated, and strength centrality—which demonstrated adequate stability—was used as the primary index to identify core symptoms.

**Results:**

Exploratory factor analysis revealed five distinct symptom clusters: emotional symptoms, fluid and electrolyte imbalance symptoms, gastrointestinal symptoms, uremic toxins symptoms, and sleep disturbance symptoms. Network analysis demonstrated that muscle cramps exhibited the highest strength centrality (1.758), followed by nervousness (1.332) and worrying (1.238), identifying muscle cramps as the most central symptom within the network structure. The correlation stability (CS) coefficient of strength centrality was 0.749, indicating adequate stability, whereas closeness and betweenness centralities were unstable (CS-coefficient = 0.00) and were therefore not interpreted.

**Conclusion:**

MHD patients experience multiple co-occurring symptom clusters, with muscle cramps identified as the most central symptom in the symptom network and fatigue representing the most severe and second most prevalent symptom. These findings highlight the importance of prioritizing interventions targeting muscle cramps, fatigue, and other high-strength symptoms. Healthcare professionals should develop tailored, symptom-specific management strategies to effectively alleviate symptom burden and enhance clinical outcomes in MHD patients.

## Introduction

End-stage kidney disease (ESKD), the fifth stage of chronic kidney disease (CKD), represents the common clinical manifestation of various renal diseases progressing to their terminal phase (Li, Jiang & Lin, 2014). In recent years, the incidence of ESKD in China has been accelerating. Among renal replacement therapies, hemodialysis (HD) is the most prevalent modality, with approximately 80% of ESKD patients in most countries opting for this treatment (Thurlow et al., 2021). As of December 2023, the total number of maintenance hemodialysis (MHD) patients in China reached 910,000, with projections indicating continued growth (Medical Administration Department of National Health Commission of China, 2024).

Although MHD can partially improve patients’ physiological function, it cannot fully replicate renal function. Studies have demonstrated that MHD patients commonly experience substantial somatic symptom burdens, including fatigue and decreased appetite (Wang, Zhang & Xiao, 2024; Le Leu et al., 2023). The cumulative effect of multiple symptoms impairs sleep quality and overall quality of life in these patients (Wang et al., 2016). Additionally, factors such as high treatment costs and inability to maintain employment may precipitate psychological distress, including anxiety and depression. Therefore, further research is urgently needed to develop more effective symptom management strategies for alleviating patient symptoms.

With advances in symptom management research, Dodd et al. (2001) first introduced the concept of symptom clusters in 2001, defining it as a constellation of three or more interrelated symptoms that may or may not share a common etiological mechanism. Due to variations in study populations and statistical methodologies, consensus regarding the minimum number of symptoms constituting a cluster remains elusive. However, it is generally agreed that symptom clusters comprise at least two or more relatively stable symptoms. As a long-term therapeutic modality for chronic kidney disease, MHD has garnered increasing research attention on symptom clusters in recent years. Currently, most studies (Wang, Zhang & Xiao, 2024) focus on the composition and influencing factors of symptom clusters at a single time point, lacking specificity. According to the latest American expert consensus (Miaskowski et al., 2017), research focusing solely on symptom clusters is insufficient for achieving more precise clinical symptom intervention and management, highlighting the urgent need for enhanced precision in symptom management.

In recent years, network analysis (NA) has been increasingly applied to symptom cluster research with the development of statistical techniques (Kuang et al., 2024). NA represents the intrinsic characteristics among symptoms through “nodes” and “edges,” demonstrating symptom correlations, with thicker connecting lines indicating stronger associations. Through network-formatted visualization models, the complex interrelationships among symptoms are displayed more intuitively. Combined with clinical expertise, this approach facilitates the identification of core symptoms within the network and exploration of potential intervention targets for dialysis-related symptoms.

Therefore, this study employs symptom network analysis to investigate symptom interactions in MHD patients, with the following objectives: (1) to identify symptoms and extract symptom clusters in MHD patients; (2) to construct a symptom network for MHD patients and identify core symptoms. This multicenter survey forms part of a broader research program on symptom experience in MHD patients in Fujian Province, China. A related analysis of the same cohort, which identified symptom clusters and characterized their 1-year longitudinal trajectories using latent class growth modeling, has been published elsewhere (Liu et al., 2026). The present study addresses a distinct research question: using the cross-sectional baseline data of this cohort, it applies exploratory factor analysis and network analysis to map fine-grained inter-symptom relationships and to identify central symptoms as candidate intervention targets, which was not examined in our previous report.

## Methods

### Subjects and methods

#### Study subjects

In June 2022, 510 hemodialysis patients were recruited using convenience sampling from hemodialysis centers at three tertiary Grade A general hospitals in Fujian Province and seven secondary medical consortium-affiliated hemodialysis centers closely collaborating with these institutions.

(1) Inclusion criteria: age ≥18 years; receiving regular hemodialysis at one of the 10 dialysis centers for ≥3 months, three times per week for 4 h per session; normal language and communication abilities; informed consent and voluntary participation.
(2) Exclusion criteria: conditions precluding study completion, including psychiatric disorders, coma, or severe cognitive dysfunction; patients who would not maintain dialysis at the study centers after discharge; patients with severe complications (*e.g*., cerebrovascular sequelae, respiratory failure).
(3) Dropout criteria: death or treatment withdrawal during the study period; voluntary withdrawal from the study for any reason.

The study was approved by the Medical Ethics Committee of the Second Affiliated Hospital of Fujian Medical University (Approval No. [2022] Fujian Medical Second Hospital Ethics Review No. 159). All research methods adhered to relevant guidelines and the Declaration of Helsinki.

#### Research instruments

##### General information questionnaire

A self-designed general information questionnaire was employed to collect demographic and clinical characteristics, including age, gender, marital status, educational level, medical insurance status, and primary disease etiology.

##### Dialysis symptom index scale for hemodialysis patients

This study employed the adapted Dialysis Symptom Index (DSI) scale developed by Hao (2016), based on the original DSI created by Weisbord et al. (2004) for hemodialysis patients, with two additional dimensions: symptom frequency and severity. The scale comprises 30 items and one open-ended question. Each item encompasses four dimensions: symptom presence (dichotomous response), symptom frequency, symptom severity (four-point rating scale), and symptom burden (five-point rating scale). The scale was translated into Chinese in 2013, with the Chinese version demonstrating a content validity index of 0.90, a Cronbach’s α coefficient of 0.87, and test-retest reliability of 0.92 (Zhou, 2013). The adapted DSI scale exhibited an overall Cronbach’s α coefficient of 0.983, with subscale Cronbach’s α coefficients of 0.943 for frequency, 0.948 for severity, and 0.945 for burden, indicating excellent internal consistency and suitability for symptom assessment in MHD patients.

#### Sample size calculation

The DSI scale employed in this study encompasses 30 symptoms. Sample size requirements dictate 5–10 times the number of variables, with an additional 20% allowance for potential attrition. Accordingly, the final sample size was 510 participants.

#### Quality control

Prior to survey initiation, researchers conducted one-on-one standardized training for investigators at each center. Liaison officers at participating hospitals were designated as coordinators. Standardized instructions were employed across all questionnaires, with investigators maintaining communication with patients and providing explanations throughout the process. Of the 510 questionnaires distributed, 502 were valid and included in the final analysis, yielding an effective response rate of 98.43%; the remaining eight questionnaires were excluded because they contained substantial missing data that precluded analysis.

#### Data collection procedures

Effective communication was established with subjects meeting the inclusion and exclusion criteria. The purpose, significance, and procedures of the study were explained to participants, who were informed of their right to voluntarily complete the questionnaires and withdraw at any time. Written informed consent was obtained from all participants. Researchers distributed questionnaires during dialysis sessions using standardized instructions. When participants encountered items requiring clarification, investigators provided immediate interpretation. Participants independently completed the questionnaires on their dialysis days. Upon collection, questionnaires were immediately examined for completeness, and participants were asked to complete any missing items.

#### Statistical methods

Statistical analyses were performed using SPSS Statistics 22.0 and R software (version 4.3.1; R Core Team, 2023). Network models were constructed using R software, with network visualization conducted using the qgraph (version 1.9.8) and bootnet (version 1.5.6) packages.

Descriptive analysis: Categorical variables were described using frequencies and percentages. Continuous variables meeting normality assumptions were expressed as mean ± standard deviation ( $ {{\bar {\rm x}} \pm s}$), with means serving as auxiliary evaluation tools.

Exploratory factor analysis (EFA): Following the statistical methodology of Kim et al. (2008), EFA was performed on symptoms to identify symptom clusters. Prior to factor analysis, the Kaiser-Meyer-Olkin (KMO) measure was used to test partial correlations among variables, with values ranging from 0 to 1; values closer to 1 indicate stronger inter-variable correlations. Bartlett’s test of sphericity was employed to verify inter-variable correlations, with *P* < 0.05 indicating suitability for EFA. Principal component analysis with varimax orthogonal rotation was utilized to extract common factors (symptom clusters). Symptom clusters were subsequently labeled based on constituent symptom characteristics, relevant literature, and clinical expertise.

Network analysis: Network models were constructed using R software, with visualization performed using the qgraph and bootnet packages. The symptom network comprises nodes and edges, where nodes represent individual symptoms and edges (connecting lines between nodes) represent correlations between symptoms; thicker lines indicate stronger associations. Central indices were identified using the networktools package (version 1.5.1]) in R. Network centrality indices primarily include three measures: strength centrality, closeness centrality, and betweenness centrality. Strength centrality represents the sum of edge weights connecting a node to all directly linked nodes, reflecting the node’s position within the network. Betweenness centrality indicates the number of shortest paths passing through a node, measuring its importance in connecting other nodes. Closeness centrality is the reciprocal of the average shortest path length between nodes, reflecting a node’s proximity to other nodes (Kuang et al., 2024).

In symptom networks, strength centrality is the most stable index (Zhu et al., 2023). Therefore, this study selected strength as the primary criterion for identifying core symptoms, facilitating identification of key intervention targets for healthcare professionals (Yu et al., 2023). The bootnet package was utilized to assess centrality index stability following sample reduction, with calculation of the correlation stability coefficient (CS-coefficient). The CS-coefficient should be at least 0.25, preferably exceeding 0.50 (Epskamp, Borsboom & Fried, 2018). Centrality indices with CS-coefficients below 0.25 were considered unstable and were not interpreted (Epskamp, Borsboom & Fried, 2018). Statistical significance was set at *P* < 0.05.

## Results

### General characteristics

A total of 502 MHD patients were included in this study. The majority were male (68.3%). Patient ages ranged from 18 to 88 years, with a mean age of 52.03 ± 12.16 years. Detailed demographic characteristics are presented in Table 1.

**Table 1:** General characteristics of hemodialysis patients (*n* = 502).

Variable	Category	Frequency (*n*)	Percentage (%)
Gender	Male	343	68.3
	Female	159	31.7
Age (years)	18–30	16	3.2
	31–40	80	15.9
	41–50	133	26.5
	51–60	149	29.7
	>60	124	24.7
Dialysis vintage (years)	<1	204	40.6
	1–3	128	25.8
	4–5	44	8.8
	6–10	96	19.1
	>10	30	6
Marital status	Married	442	88
	Single	35	7
	Divorced	7	1.4
	Widowed	18	3.6
Educational level	Junior high school or below	325	64.7
	High school or technical secondary school	130	25.9
	High school or technical secondary school	47	9.4
Medical insurance type	Employee medical insurance	399	75.9
	New rural cooperative medical scheme	99	15.7
	Self-pay	4	0.8
Primary disease	Chronic glomerulonephritis	255	50.8
	Hypertensive nephropathy	73	14.5
	Diabetic nephropathy	120	23.9
	Polycystic kidney disease	11	2.2
	Other	43	8.6
Monthly household income *per capita* (CNY)	<3,000	207	41.2
	3,001–5,000	194	38.6
	>5,000	111	22.1

**Note:**

CNY, Chinese Yuan.

### Symptom prevalence in MHD patients

Results revealed that the five most prevalent symptoms in MHD patients were difficulty falling asleep (53.19%), fatigue or tiredness (49.40%), frequent awakening (47.21%), itching (39.44%), and muscle cramps (37.05%). The five symptoms with the highest severity scores were fatigue or tiredness (1.55 ± 1.68), difficulty falling asleep (1.16 ± 1.26), dry mouth (1.03 ± 1.46), muscle cramps (0.96 ± 1.47), and itching (0.89 ± 1.38). Detailed results are presented in Table S1.

### Symptom cluster extraction in MHD patients

According to previous research, symptoms with low prevalence cannot reflect the key characteristics of symptom clusters (Kim et al., 2008). A total of 21 symptoms were extracted for symptom cluster analysis: constipation, nausea, vomiting, decreased appetite, muscle cramps, lower extremity swelling, shortness of breath, fatigue or tiredness, cough, dry mouth, bone or joint pain, muscle pain, dry skin, itching, worrying, nervousness, difficulty falling asleep, easy awakening, irritability, feeling sad, and anxiety. The Kaiser-Meyer-Olkin (KMO) measure yielded a value of 0.77, and Bartlett’s test of sphericity showed $\chi^2$ = 4,555.76 (df = 210), *P* < 0.001, indicating correlations among symptoms and suitability for factor analysis.

Principal component analysis with varimax rotation was employed to conduct exploratory factor analysis to identify symptom clusters with common characteristics, based on the following criteria: ① eigenvalue ≥1.0; ② each factor contains at least two symptoms. Factor analysis requires that the total cumulative variance contribution rate should be ≥50%. In this study, factor analysis of 21 symptoms in MHD patients revealed five symptom clusters with a cumulative variance contribution rate of 52.71%. Considering the clinical interpretability of the symptom clusters, five symptom clusters were ultimately included. They were designated as follows: Factor 1, emotional symptom cluster, including worrying, nervousness, irritability, feeling sad, and anxiety; Factor 2, fluid and electrolyte imbalance symptom cluster, including muscle cramps, lower extremity swelling, shortness of breath, cough, bone or joint pain, and muscle pain; Factor 3, gastrointestinal symptom cluster, including constipation, nausea, vomiting, and decreased appetite; Factor 4, uremic toxin symptom cluster, including fatigue or tiredness, dry mouth, dry skin, and itching; Factor 5, sleep disturbance symptom cluster, including difficulty falling asleep and frequent awakening. The factor loadings for the five factors and their associated symptoms are presented in Table 2.

**Table 2:** Symptom clusters and factor loadings in MHD patients.

Symptom	Factor 1	Factor 2	Factor 3	Factor 4	Factor 5
Constipation	–	–	0.873	–	–
Nausea	–	–	0.761	–	–
Vomiting	–	–	0.782	–	–
Decreased appetite	–	–	0.813	–	–
Muscle cramps	–	0.855	–	–	–
Lower extremity swelling	–	0.749	–	–	–
Shortness of breath	–	0.555	–	–	–
Fatigue or tiredness	–	–	–	0.658	–
Cough	–	0.726	–	–	–
Dry mouth	–	–	–	0.691	–
Bone or joint pain	–	0.691	–	–	–
Muscle pain	–	0.571	–	–	–
Dry skin	–	–	–	0.452	–
Itching	–	–	–	0.546	–
Worrying	0.844	–	–	–	–
Nervousness	0.833	–	–	–	–
Difficulty falling asleep	–	–	–	–	0.609
Frequent awakening	–	–	–	–	0.658
Irritability	0.771	–	–	–	–
Feeling sad	0.724	–	–	–	–
Anxiety	0.779	–	–	–	–
Eigenvalue	3.18	2.95	2.65	1.43	0.85
Variance contribution rate (%)	15.15	14.06	12.61	6.83	4.07
Cumulative variance contribution rate (%)	15.15	29.21	41.81	48.65	52.71
Model explanation rate	28.73	26.68	23.91	12.96	7.71
Cumulative explanation rate	28.73	55.41	79.33	92.29	100.00

**Note:**

Factor 1 = Emotional symptom cluster; Factor 2 = Fluid and electrolyte imbalance symptom cluster; Factor 3 = Gastrointestinal symptom cluster; Factor 4 = Uremic toxin symptom cluster; Factor 5 = Sleep disturbance symptom cluster. “–” indicates factor loading <0.40.

### Network analysis of symptoms in MHD patients

**Figure 1:**
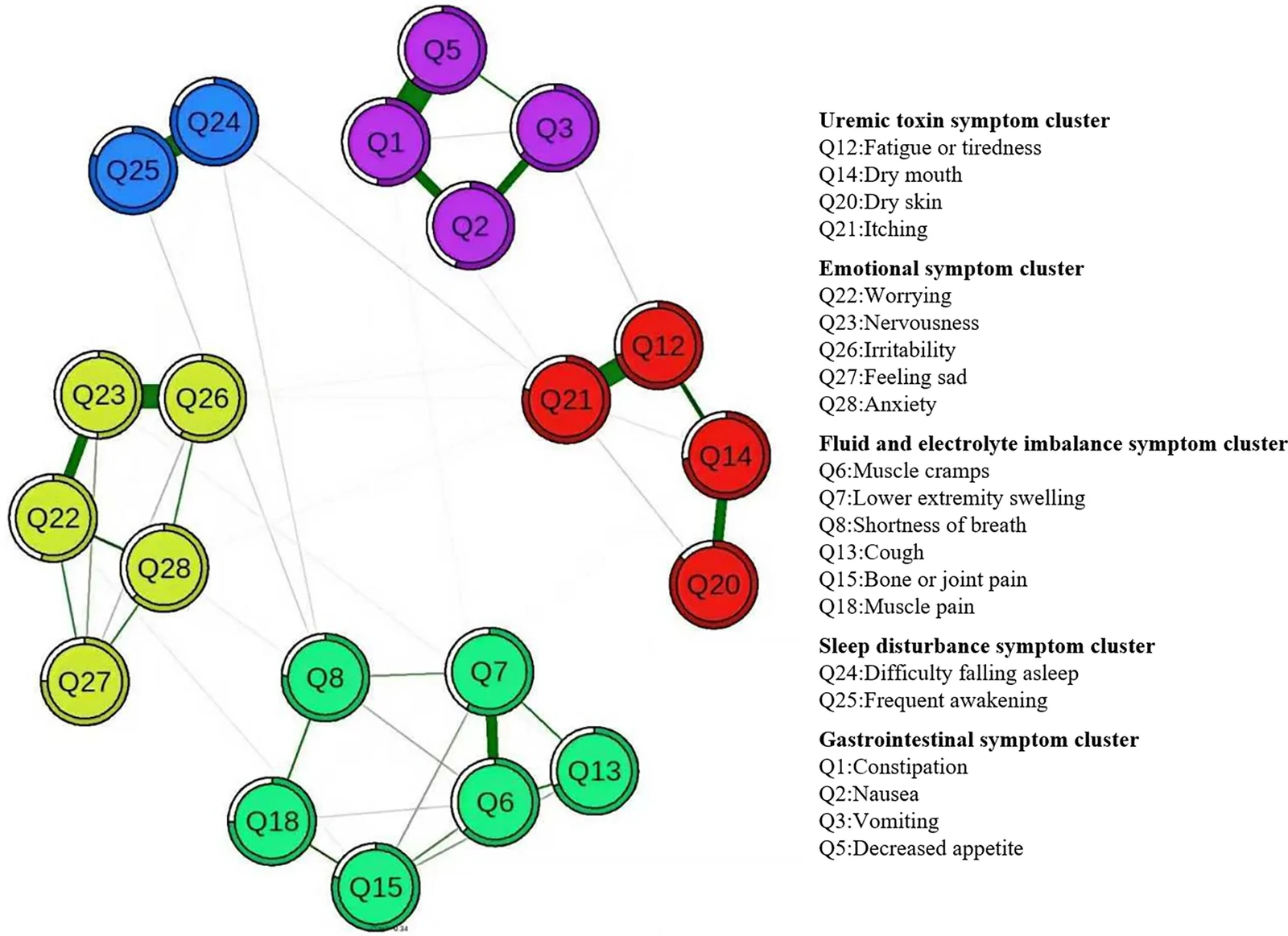
Symptom network estimation in MHD patients. “Nodes” represent symptoms, and “edges” represent the relationships between symptoms.

**Figure 2:**
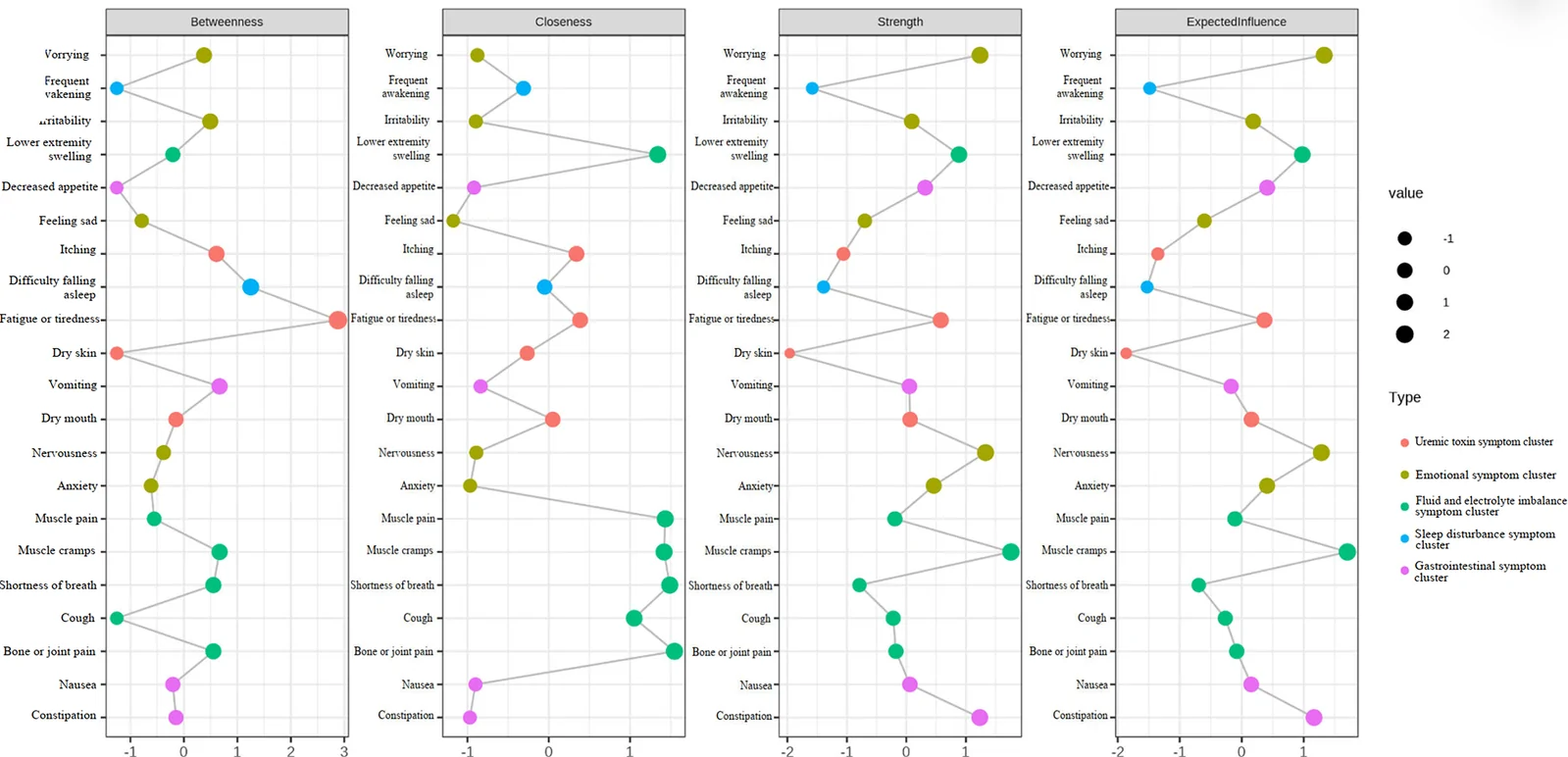
Centrality indices of individual symptoms in the symptom network of MHD patients. Betweenness = betweenness centrality; Closeness = closeness centrality; Strength = strength centrality. The x-axis represents the specific values of centrality indices; the y-axis represents the individual symptoms experienced by maintenance hemodialysis patients.

As illustrated in Fig. 1, the five symptom clusters in MHD patients are represented by different colors. Within the network, symptoms within the same cluster are connected by dark-colored, thick edges, indicating positive correlations among intra-cluster symptoms. In contrast, inter-cluster connections are characterized by thin edges, greater distances, and lighter colors, suggesting weaker correlations between symptom clusters. The strongest edge weight in MHD patients was observed between constipation and decreased appetite (0.521), followed by the edge weight between nervousness and irritability (0.492). The corresponding centrality indices of symptom network nodes are presented in Fig. 2. Results revealed that muscle cramps had the highest strength centrality (1.758). Given the inadequate stability of closeness and betweenness centralities demonstrated in the stability analysis below (CS-coefficient = 0.00), these two indices are reported for descriptive completeness only and were not further interpreted.

According to the centrality indices of network nodes shown in Fig. 2, the three symptoms with the highest strength centrality were muscle cramps (1.758), nervousness (1.332), and worrying (1.238), indicating that muscle cramps, nervousness, and worrying are the most central symptoms in the network.

**Figure 3:**
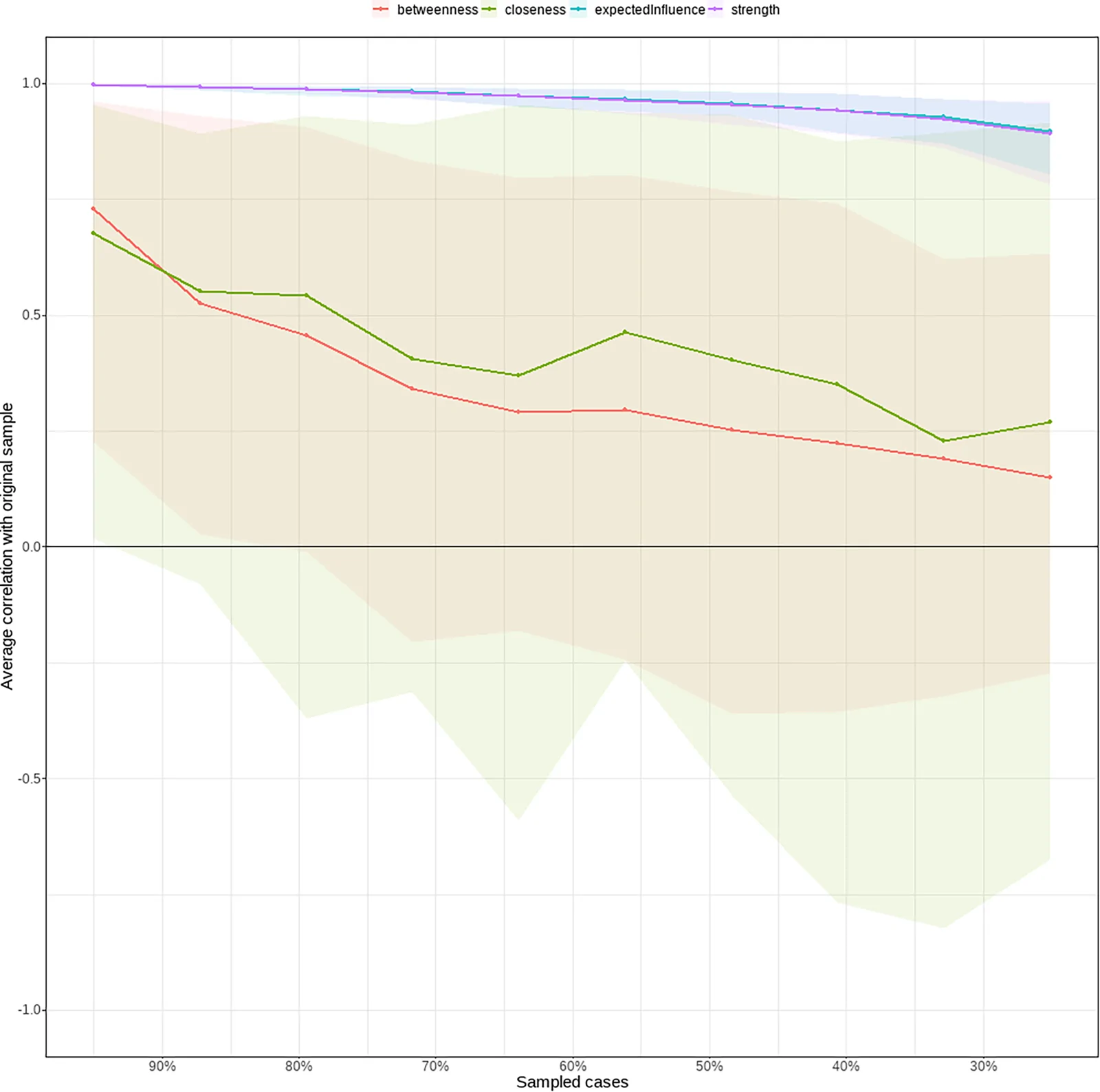
Correlation stability coefficient of the symptom network in maintenance hemodialysis patients.

Stability testing of centrality indices: The correlation stability analysis of the symptom cluster network in MHD patients is shown in Fig. 3. In this study, the correlation stability coefficients (CS-coefficients) for strength, expected influence, closeness centrality, and betweenness centrality were 0.749, 0.749, 0.00, and 0.00, respectively. As illustrated in Fig. 3, the stability coefficients of closeness centrality and betweenness centrality decreased with sample reduction. According to established guidelines, centrality indices with CS-coefficients below 0.25 should not be interpreted (Epskamp, Borsboom & Fried, 2018); therefore, closeness and betweenness centralities were excluded from interpretation, and core symptoms were identified on the basis of strength centrality (CS-coefficient = 0.749), which demonstrated the highest stability among the centrality indices.

**Figure 4:**
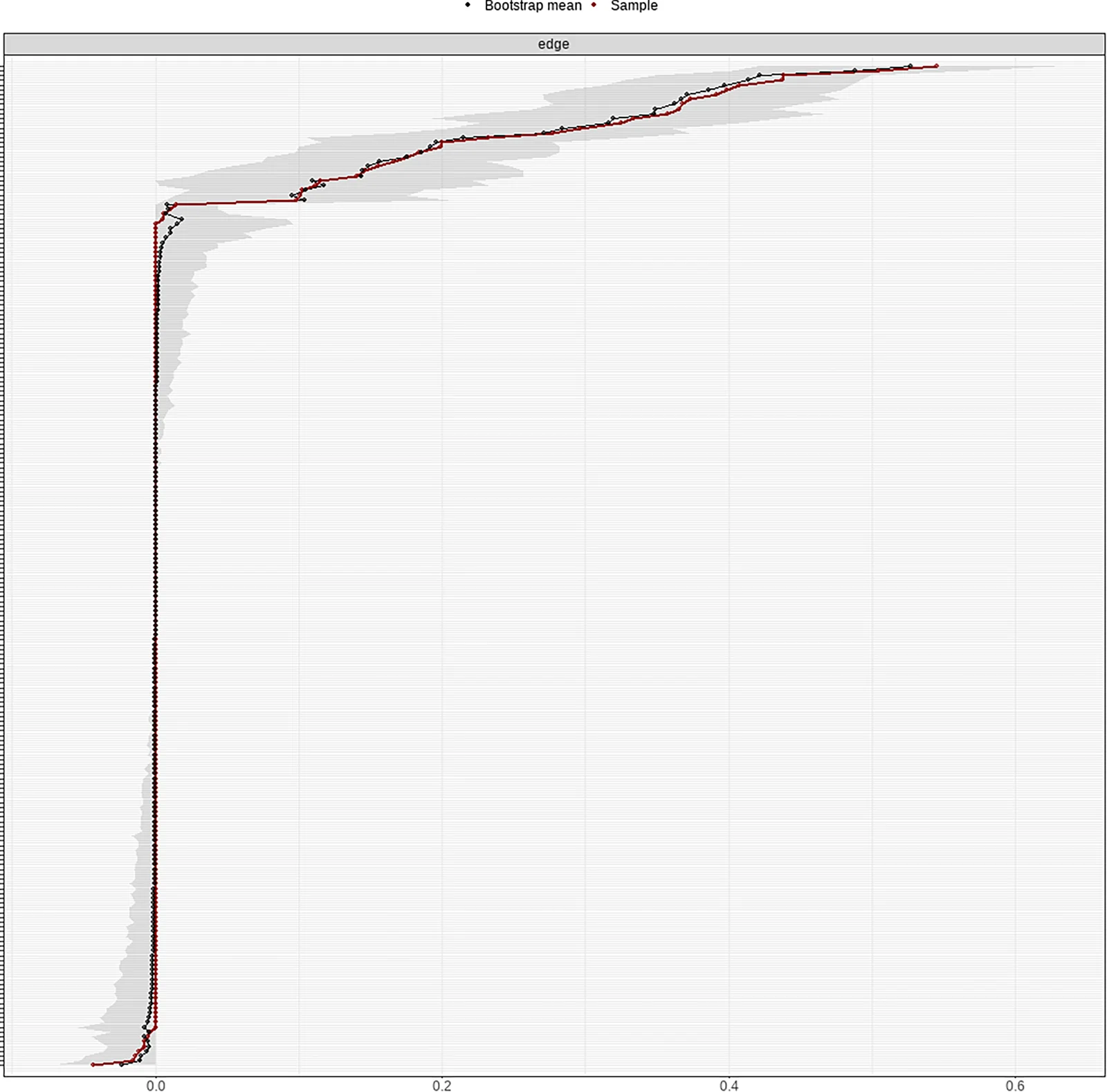
Bootstrapped edge weight parameters of the symptom network.

Accuracy testing of edge weights: The bootstrap analysis results for edge weights in the symptom network are presented in Fig. 4. The narrow 95% confidence intervals (gray areas) of the edge weights indicate good accuracy of the network.

## Discussion

### Analysis of general characteristics of MHD patients

This study enrolled 502 MHD patients, with individuals aged over 50 years accounting for a substantial proportion. The mean age was 52.03 ± 12.16 years, indicating that the MHD population predominantly consisted of middle-aged and elderly individuals. Patients with a dialysis vintage of less than 5 years constituted the majority (75.2%), with a median dialysis vintage of 42 months, consistent with findings from the national hemodialysis case information system regarding patient demographics (Chen, 2018). The longest dialysis vintage exceeded 22 years, while the shortest was exactly 3 months.

### Symptom profile of MHD patients

In this study, symptom prevalence ranged from 12.35% to 53.19%, with difficulty falling asleep being the most prevalent (53.19%), followed by fatigue or tiredness (49.40%). However, these rates were lower than those reported in similar domestic studies (Hao, 2016; Zhai et al., 2023). This discrepancy may be attributed to bias introduced by convenience sampling and to the fact that the majority of patients in this study (40.6%) had a dialysis vintage of less than 1 year, whereas MHD patients with longer dialysis vintage face higher risks of developing complications such as sleep disorders (Vendeville, Mucsi & Molnar, 2025). Difficulty falling asleep in MHD patients is primarily associated with factors such as skin itching and restless legs syndrome (Lyons, 2024), and chronic poor sleep quality can further cause or exacerbate fatigue. Additionally, multiple factors including comorbidities, activities of daily living, ultrafiltration rate, and post-dialysis blood pressure can influence the degree of fatigue in patients (Bossola et al., 2023). The prevalence of decreased libido and difficulty with arousal in this study was substantially lower than the 65.2% and 56.5% reported in previous studies (Zhang et al., 2024), which may be related to the fact that most participants were from rural townships with relatively low educational levels (64.7% had middle school education or below), coupled with conservative attitudes toward sexuality that may have prevented accurate symptom reporting.

### Symptom cluster analysis in MHD patients

This study identified five symptom clusters, which were named based on existing research and clinical experience: emotional symptom cluster, uremic symptom cluster, fluid and electrolyte imbalance symptom cluster, gastrointestinal symptom cluster, and sleep disturbance symptom cluster.

#### Emotional symptom cluster

The emotional symptom cluster comprised five symptoms: worrying, nervousness, irritability, feeling sad, and anxiety. This finding is highly consistent with previous studies (Hao, 2016; Zhou et al., 2023), demonstrating that emotional symptom clusters are universally present in MHD patients. The clustering of various emotion-related symptoms exerts a greater impact on patients than individual symptoms alone. Although different studies employ varying nomenclature—such as psychological or affective symptom clusters—these clusters consistently include two or more of the following: worrying, nervousness, irritability, feeling sad, and anxiety. Some studies have incorporated sleep disturbance symptoms into the emotional symptom cluster. Related research has shown that the prevalence of depressive symptoms among dialysis patients remains considerably high (Elezi et al., 2023). This may be attributed to the burden of long-term, repeated hospital visits for treatment, strict dietary and fluid restrictions, and the development of multiple comorbidities during the later stages of maintenance dialysis. These factors collectively contribute to numerous physical and psychological symptoms that severely compromise patients’ quality of life. Therefore, healthcare professionals should prioritize the assessment of patients’ psychological states in daily clinical practice and implement targeted psychological interventions to alleviate the burden of emotional symptom clusters.

#### Fluid and electrolyte imbalance symptom cluster

The fluid and electrolyte imbalance symptom cluster indicates the presence of electrolyte disturbances and fluid imbalance in MHD patients, encompassing muscle cramps, lower extremity edema, shortness of breath, cough, bone or joint pain, and muscle pain. Since most MHD patients undergo dialysis every other day, poor fluid control in some patients readily leads to lower extremity edema, which can subsequently trigger left-sided heart failure. Research suggests that water-sodium retention and electrolyte disturbances in MHD patients exert multifaceted effects on the body, including increased cardiovascular burden, musculoskeletal dysfunction, and impaired respiratory function (Zoccali et al., 2017). Therefore, healthcare professionals should assist patients with individualized fluid management, strengthen education on interdialytic weight gain control, regularly monitor electrolyte levels, and adjust dialysis prescriptions based on laboratory results to reduce adverse outcomes associated with this symptom cluster and improve patient outcomes.

#### Gastrointestinal symptom cluster

The gastrointestinal symptom cluster is a common symptom cluster in MHD patients, potentially related to long-term dietary restrictions and inadequate dialysis. In this study, the symptom cluster included constipation, nausea, vomiting, and decreased appetite, which is highly consistent with domestic and international research on gastrointestinal symptom clusters (Zhou, 2013; Al Awadhi et al., 2025; Moore et al., 2022; Ng et al., 2020). The internal composition of gastrointestinal symptom clusters varies slightly across studies due to differences in assessment tools and statistical methods. Some studies have identified gastrointestinal symptom clusters containing only nausea and vomiting, while others also include decreased appetite, constipation, or diarrhea. A 12-month follow-up study of dialysis patients conducted by Hong Kong scholars found that nausea and vomiting were the predominant symptoms (Ng et al., 2020).

The pathophysiology of gastrointestinal symptom clusters is complex, potentially involving uremic toxin accumulation, gastrointestinal hormone dysregulation, gut microbiota dysbiosis, and dialysis-related factors (Al Awadhi et al., 2025; Vaziri et al., 2013). MHD patients often experience inadequate nutritional intake due to poor appetite or excessive dietary restrictions, which subsequently reduces dialysis tolerance and treatment effectiveness, creating a vicious cycle of “malnutrition-symptom exacerbation-further malnutrition” that severely impedes physical recovery. Therefore, healthcare professionals should strengthen systematic assessment of gastrointestinal symptom clusters in MHD patients and implement multidimensional intervention strategies: ① promptly optimize dialysis prescriptions based on symptom severity to ensure dialysis adequacy; ② provide individualized nutritional counseling, improve food preparation methods, and enhance dietary palatability within restriction parameters; ③ strengthen nutritional education regarding dialysis diet to correct misconceptions about dietary restrictions; ④ consult with nutritionists when necessary to develop targeted nutritional support plans. Through comprehensive management, these approaches can promote nutritional recovery, improve dialysis quality, and enhance patients’ quality of life.

#### Uremic symptom cluster

The uremic symptom cluster identified in this study comprised fatigue or tiredness, dry mouth, dry skin, and itching, with fatigue and pruritus demonstrating notably high prevalence. Previous studies (Zhou, 2013; Al Awadhi et al., 2025) have documented that uremic toxin-related symptoms in MHD patients encompass fatigue or weakness, headache, dizziness, numbness in feet, muscle cramps, and joint pain. Some investigations (You et al., 2022b) have incorporated sleep disturbances (difficulty falling asleep and easy awakening) into this cluster, while Amro et al. (2015) observed uremic symptoms co-occurring with chest pain. These discrepancies indicate poor consistency in uremic symptom cluster composition across studies, primarily attributable to heterogeneity in assessment instruments and statistical methodologies.

Despite compositional variations, the uremic symptom cluster is widely recognized as the most clinically significant symptom cluster in CKD patients, with its severity stemming from synergistic pathophysiological mechanisms. The accumulation of small- and middle-molecular uremic toxins, anemia, secondary hyperparathyroidism, chronic low-grade inflammation, and dialysis inadequacy collectively drive the development and progression of this symptom cluster (Al Awadhi et al., 2025; Vanholder et al., 2003). More critically, the uremic symptom cluster not only severely impairs patients’ physical and psychological well-being and quality of life but also substantially increases mortality risk. A prospective cohort study demonstrated that patients with uremic symptom clusters exhibited more than twice the mortality risk compared to those with other symptom clusters (Amro et al., 2015), potentially reflecting greater toxin burden and inadequate dialysis.

Given the high prevalence and adverse prognosis associated with uremic symptom clusters, clinical management should prioritize this cluster as a key intervention target. A multidimensional strategy is recommended: ① regularly assess and optimize dialysis adequacy (Kt/V ≥1.2), extending dialysis duration or increasing frequency when necessary; ② select high-flux or high-cutoff dialyzers based on individual toxin clearance requirements to enhance middle-molecular toxin removal; ③ actively manage related complications, including anemia correction (target hemoglobin 110–120 g/L) and secondary hyperparathyroidism control (target iPTH 150–300 pg/mL); ④ implement stepwise treatment for refractory pruritus, progressing from skin moisturization and dialysate calcium optimization to gabapentinoid or κ-opioid receptor agonist therapy when necessary. Through systematic interventions to reduce uremic toxin burden, substantial improvements in symptom burden, quality of life, and long-term prognosis can be anticipated.

#### Sleep disturbance symptom cluster

The sleep symptom cluster identified in this study comprised difficulty falling asleep and early awakening, which manifested independently of other symptom clusters. These symptoms ranked first and third in prevalence, affecting 53.19% and 47.21% of patients, respectively. Epidemiological data suggest that approximately 41–85% of maintenance hemodialysis (MHD) patients experience sleep disturbances (Davydov et al., 2023). Although the underlying mechanisms remain incompletely understood, sleep disorders in this population likely arise from the convergence of multiple chronic stressors. Physical burdens—including pruritus, pain, and restless legs syndrome—compound psychological distress such as anxiety and depression, collectively precipitating sleep disturbance symptom clusters. Left unaddressed, these sleep disorders can exacerbate fatigue and emotional symptoms, creating a vicious cycle that substantially compromises quality of life and long-term survival in MHD patients (Lyons, 2024). Given these far-reaching consequences, sleep disturbances in hemodialysis populations warrant heightened clinical attention and targeted intervention strategies.

### Core symptoms in the symptom network of MHD patients

Network centrality analysis based on strength centrality—the only centrality index with adequate stability in this study—revealed that muscle cramps (1.758), nervousness (1.332), and worrying (1.238) exhibited the highest strength centrality, indicating their extensive direct connections with neighboring symptoms within the network. In contrast, the closeness and betweenness centrality estimates were unstable (CS-coefficient = 0.00) and were therefore not interpreted.

On the basis of its stable strength centrality, muscle cramps emerged as the most central symptom in the symptom network of MHD patients. Muscle cramps, affecting 37.05% of patients, ranked among the five most prevalent symptoms despite lower frequency compared to similar studies (Zhang et al., 2024). This symptom typically arises from excessive ultrafiltration, intradialytic hypotension, hypocalcemia, and secondary carnitine depletion (Mantilla-Manosalva et al., 2024). Given the excruciating pain patients endure during episodes and its central network position, prioritizing evidence-based interventions for muscle cramps represents a crucial strategy for symptom management and complication reduction.

Although fatigue was not among the three symptoms with the highest strength centrality, it warrants particular clinical attention on the basis of its symptom burden: fatigue presented with the highest severity score (1.55 ± 1.68) and the second-highest prevalence (49.40%) in this cohort, and it constitutes the cardinal manifestation of the uremic symptom cluster—findings consistent with prior research on core symptoms (Zhai et al., 2023). In other MHD cohorts, the prevalence of fatigue has been reported to reach 64% (Vendeville, Mucsi & Molnar, 2025). Mechanistically, fatigue in MHD patients stems from multifactorial origins: treatment-related parameters including ultrafiltration volume, dialysis vintage, intradialytic hypotension, dialyzer membrane characteristics, and dialysate sodium concentration (You et al., 2022a; Zu et al., 2020); and patient-specific factors such as age, comorbidity burden, psychological distress, and sleep fragmentation (Zhang et al., 2025). Evidence suggests bidirectional relationships wherein fatigue both triggers and amplifies anxiety, sleep disturbances, and other symptoms (Huang, Li & Zeng, 2025). Consequently, targeted interventions addressing fatigue may yield improvements across multiple symptom domains.

From a clinical translation perspective, caution is warranted when extrapolating these network findings to intervention design. Because the closeness and betweenness centrality estimates were unstable in this study, it cannot be determined whether targeting a central symptom such as muscle cramps would produce “global” improvements across the entire symptom network or merely “local” relief of its directly connected neighbors (*e.g*., other fluid and electrolyte imbalance symptoms). Moreover, the cross-sectional, contemporaneous nature of the estimated network precludes causal inference regarding symptom-to-symptom influences, and both muscle cramps and fatigue are frequently refractory symptoms in MHD patients. The present network findings should therefore be regarded as hypothesis-generating: they prioritize candidate intervention targets—most robustly muscle cramps, given its high and stable strength centrality—whose modification should be tested in longitudinal and interventional studies before network-wide benefits can be claimed.

Symptom-specific associations revealed that constipation demonstrated the strongest correlation with decreased appetite, while nervousness and irritability exhibited the tightest linkage within the emotional symptom domain. Notably, closeness and betweenness centralities demonstrated inadequate stability in this analysis (CS-coefficient = 0.00), indicating vulnerability to sample fluctuations that may compromise rank consistency and interpretability; rank-based interpretations of these two indices were therefore avoided, and strength centrality was prioritized when identifying core symptoms.

## Limitations

This study has several limitations. First, the cross-sectional design precludes causal inference regarding the directional relationships among symptoms; the estimated network represents contemporaneous associations, and longitudinal studies are needed to clarify temporal and potentially causal pathways. Second, convenience sampling from ten centers in a single region may limit the generalizability of the findings. Third, dialysis adequacy indicators (*e.g*., Kt/V) were not collected in this questionnaire-based survey; because dialysis adequacy may influence uremic symptoms, future studies should incorporate Kt/V and related laboratory indicators to account for its potential confounding effect. Fourth, closeness and betweenness centralities were unstable (CS-coefficient = 0.00) and could not be interpreted; the identification of core symptoms therefore relied on strength centrality alone. Finally, symptoms were assessed by self-report, which may be subject to recall and reporting biases.

## Practical application

Muscle cramps functions as the most central node in the symptom network of maintenance hemodialysis patients, and fatigue constitutes the most severe and second most prevalent symptom. Clinicians should prioritize systematic screening and evidence-based management of these symptoms—including ultrafiltration optimization, dialysate adjustment, and psychological support. Given the cross-sectional design and the instability of bridge-related centrality indices, network-informed multidisciplinary interventions targeting high-strength symptoms should be evaluated in longitudinal and interventional studies to determine whether they reduce the overall symptom burden and enhance quality of life.

## Conclusion

This study systematically characterized the symptom burden in patients undergoing maintenance hemodialysis (MHD). Prevalence analysis revealed the five most common symptoms: difficulty falling asleep, fatigue or tiredness, easy awakening, itching, and muscle cramps. When assessed by severity scores, the most distressing symptoms were fatigue or tiredness, difficulty falling asleep, dry mouth, muscle cramps, and itching. Through cluster analysis, we identified five distinct symptom clusters: the emotional symptom cluster, fluid and electrolyte imbalance symptom cluster, gastrointestinal symptom cluster, uremic toxin symptom cluster, and sleep disturbance symptom cluster. Network analysis based on strength centrality—the only stable centrality index—further revealed that muscle cramps occupied the most central position within the symptom network, followed by nervousness and worrying, suggesting their pivotal roles in symptom interactions. Fatigue, although not the most central node, represented the most severe and second most prevalent symptom. These findings underscore the importance of prioritizing muscle cramps, fatigue, and other high-strength symptoms in clinical management. Healthcare professionals should develop evidence-based, targeted interventions focusing on these symptoms to effectively reduce symptom burden and enhance quality of life in MHD patients.

## Supplemental Information

10.7717/peerj.21741/supp-1 Supplemental Information 1 Raw Data.

10.7717/peerj.21741/supp-2 Supplemental Information 2 Codebook.

10.7717/peerj.21741/supp-3 Supplemental Information 3 Symptom Prevalence and Severity in MHD Patients (*n* = 502).

10.7717/peerj.21741/supp-4 Supplemental Information 4 R Script.

10.7717/peerj.21741/supp-5 Supplemental Information 5 STROBE checklist.

10.7717/peerj.21741/supp-6 Supplemental Information 6 Instructions for using the scale.

## Additional Information and Declarations

### Data Availability

The following information was supplied regarding data availability:

The data and code are available in the Supplemental Files.
