# Supplementary material for: Symptom network analysis in maintenance hemodialysis patients: a multicenter study
Source: PeerJ. 2026 Sep 23;14:e21741. doi: 10.7717/peerj.21741 (PMC13615734; doi:10.7717/peerj.21741)
Supplement: Supplementary file 1 [file peerj-14-21741-s003.docx]

Supplementary Table 1 Symptom Prevalence and Severity in MHD Patients (n = 502)

| Symptom | Prevalence [n (%)] | Severity (score)  ‾x±s | Distress (score)  ‾x±s |
| --- | --- | --- | --- |
| Constipation | 146 (29.08) | 0.70±1.24 | 0.65±1.23 |
| Nausea | 127 (25.30) | 0.58±1.09 | 0.56±1.13 |
| Vomiting | 131 (26.10) | 0.55±1.16 | 0.53±1.15 |
| Diarrhea | 67 (13.35) | 0.32±0.93 | 0.30±0.90 |
| Decreased appetite | 162 (32.27) | 0.78±1.26 | 0.76±1.27 |
| Muscle cramps | 186 (37.05) | 0.96±1.47 | 0.89±1.43 |
| Lower limb swelling | 147 (29.28) | 0.76±1.33 | 0.73±1.32 |
| Shortness of breath | 77 (15.34) | 0.31±0.83 | 0.27±0.77 |
| Dizziness | 62 (12.35) | 0.27±0.78 | 0.25±0.74 |
| Restless legs | 69 (13.74) | 0.41±1.12 | 0.37±1.02 |
| Numbness or tingling in feet | 65 (12.95） | 0.25±0.75 | 0.24±0.77 |
| Fatigue or tiredness | 248 (49.40) | 1.55±1.68 | 1.43±1.64 |
| Cough | 134 (26.69) | 0.71±1.30 | 0.66±1.26 |
| Dry mouth | 189 (37.65) | 1.03±1.46 | 1.10±1.47 |
| Bone or joint pain | 136 (27.09) | 0.49±0.97 | 0.45±0.97 |
| Chest pain | 69 (13.74) | 0.28±0.80 | 0.27±0.82 |
| Headache | 66 (13.15) | 0.31±0.94 | 0.30±0.92 |
| Muscle soreness | 79 (15.74) | 0.37±0.99 | 0.35±0.96 |
| Difficulty concentrating | 67 (13.35) | 0.30±0.89 | 0.30±0.89 |
| Dry skin | 95 (18.92) | 0.30±0.74 | 0.28±0.76 |
| Itching | 198(39.44) | 0.89±1.38 | 0.82±1.32 |
| Worrying | 90 (17.93) | 0.31±0.75 | 0.30±0.78 |
| Nervousness | 97 (19.32) | 0.55±1.24 | 0.52±1.18 |
| Difficulty falling asleep | 267 (53.19) | 1.16±1.26 | 1.13±1.30 |
| Frequent awakening | 237 (47.21) | 0.94±1.18 | 0.95±1.29 |
| Irritability | 104 (20.72) | 0.62±1.32 | 0.60±1.29 |
| Feeling sad | 86 (17.13) | 0.34±0.89 | 0.32±0.89 |
| Anxiety | 88 (17.53) | 0.29±0.76 | 0.31±0.83 |
| Decreased libido | 71 (14.14) | 0.33±0.88 | 0.32±0.88 |
| Difficulty with arousal | 75 (14.94) | 0.30±0.82 | 0.29±0.82 |
