# Supplementary material for: Symptom network analysis in maintenance hemodialysis patients: a multicenter study
Source: PeerJ. 2026 Sep 23;14:e21741. doi: 10.7717/peerj.21741 (PMC13615734; doi:10.7717/peerj.21741)
Supplement: Supplementary file 2 [file peerj-14-21741-s005.docx]

STROBE Statement—checklist of items that should be included in reports of observational studies

|  | Item No. | Recommendation | Page  No. | Relevant text from manuscript |
| --- | --- | --- | --- | --- |
| **Title and abstract** | 1 | (*a*) Indicate the study’s design with a commonly used term in the title or the abstract | 1 | A Multicenter Study |
|  |  | (*b*) Provide in the abstract an informative and balanced summary of what was done and what was found | 1,2 | **Results:** Exploratory factor analysis revealed five distinct symptom clusters: emotional symptoms, fluid and electrolyte imbalance symptoms, gastrointestinal symptoms, uremic toxins symptoms, and sleep disturbance symptoms. Network analysis demonstrated that muscle cramps exhibited the highest strength centrality (1.758), fatigue showed the highest betweenness centrality (2.882), and bone/joint pain displayed the highest closeness centrality (1.547). Collectively, fatigue and muscle cramps emerged as the most central symptoms within the network structure.  **Conclusion:** MHD patients experience multiple co-occurring symptom clusters, with fatigue and muscle cramps identified as core symptoms in the symptom network. These findings highlight the importance of prioritizing interventions targeting these central symptoms. Healthcare professionals should develop tailored, symptom-specific management strategies to effectively alleviate symptom burden and enhance clinical outcomes in MHD patients. |
| Introduction | | | |  |
| Background/rationale | 2 | Explain the scientific background and rationale for the investigation being reported | 2,3 | End-stage kidney disease (ESKD), the fifth stage of chronic kidney disease (CKD), represents the common clinical manifestation of various renal diseases progressing to their terminal phase [1].Although MHD can partially improve patients' physiological function, it cannot fully replicate renal function. Studies have demonstrated that MHD patients commonly experience substantial somatic symptom burdens, including fatigue and decreased appetite [4, 5]. In recent years, network analysis (NA) has been increasingly applied to symptom cluster research with the development of statistical techniques [9]. NA represents the intrinsic characteristics among symptoms through "nodes" and "edges," demonstrating symptom correlations, with thicker connecting lines indicating stronger associations. Through network-formatted visualization models, the complex interrelationships among symptoms are displayed more intuitively. Combined with clinical expertise, this approach facilitates the identification of core symptoms within the network and exploration of potential intervention targets for dialysis-related symptoms. |
| Objectives | 3 | State specific objectives, including any prespecified hypotheses | 3 | Therefore, this study employs symptom network analysis to investigate symptom interactions in MHD patients, with the following objectives: (1) to identify symptoms and extract symptom clusters in MHD patients; (2) to construct a symptom network for MHD patients and identify core symptoms. |
| Methods | | | |  |
| Study design | 4 | Present key elements of study design early in the paper | 3,4 | In June 2022, 510 hemodialysis patients were recruited using convenience sampling from hemodialysis centers at three tertiary Grade A general hospitals in Fujian Province and seven secondary medical consortium-affiliated hemodialysis centers closely collaborating with these institutions. |
| Setting | 5 | Describe the setting, locations, and relevant dates, including periods of recruitment, exposure, follow-up, and data collection | 3,4 | In June 2022, 510 hemodialysis patients were recruited using convenience sampling from hemodialysis centers at three tertiary Grade A general hospitals in Fujian Province and seven secondary medical consortium-affiliated hemodialysis centers closely collaborating with these institutions.advanced qualifications. |
| Participants | 6 | (*a*) *Cohort study*—Give the eligibility criteria, and the sources and methods of selection of participants. Describe methods of follow-up  *Case-control study*—Give the eligibility criteria, and the sources and methods of case ascertainment and control selection. Give the rationale for the choice of cases and controls  *Cross-sectional study*—Give the eligibility criteria, and the sources and methods of selection of participants | 4 | **(1) Inclusion criteria:** age ≥18 years; receiving regular hemodialysis at one of the 10 dialysis centers for ≥3 months, three times per week for 4 hours per session; normal language and communication abilities; informed consent and voluntary participation.  **(2) Exclusion criteria:** conditions precluding study completion, including psychiatric disorders, coma, or severe cognitive dysfunction; patients who would not maintain dialysis at the study centers after discharge; patients with severe complications (e.g., cerebrovascular sequelae, respiratory failure). |
|  |  | (*b*) *Cohort study*—For matched studies, give matching criteria and number of exposed and unexposed  *Case-control study*—For matched studies, give matching criteria and the number of controls per case |  | N/A |
| Variables | 7 | Clearly define all outcomes, exposures, predictors, potential confounders, and effect modifiers. Give diagnostic criteria, if applicable |  | N/A |
| Data sources/ measurement | 8* | For each variable of interest, give sources of data and details of methods of assessment (measurement). Describe comparability of assessment methods if there is more than one group |  | N/A |
| Bias | 9 | Describe any efforts to address potential sources of bias |  | N/A |
| Study size | 10 | Explain how the study size was arrived at | 5 | The DSI scale employed in this study encompasses 30 symptoms. Sample size requirements dictate 5-10 times the number of variables, with an additional 20% allowance for potential attrition. Accordingly, the final sample size was 510 participants. |

Continued on next page

| Quantitative variables | 11 | Explain how quantitative variables were handled in the analyses. If applicable, describe which groupings were chosen and why | 5 | Prior to survey initiation, researchers conducted one-on-one standardized training for investigators at each center. Liaison officers at participating hospitals were designated as coordinators. Standardized instructions were employed across all questionnaires, with investigators maintaining communication with patients and providing explanations throughout the process. Of the 510 questionnaires distributed, 502 were retrieved, yielding an effective response rate of 98.43%. |
| --- | --- | --- | --- | --- |
| Statistical methods | 12 | (*a*) Describe all statistical methods, including those used to control for confounding | 6,7 | Statistical analyses were performed using SPSS Statistics 22.0 and R software (version 4.3.1). Network models were constructed using R software, with network visualization conducted using the qgraph and bootnet packages.  Descriptive analysis: Categorical variables were described using frequencies and percentages. Continuous variables meeting normality assumptions were expressed as mean ± standard deviation (x̄±s), with means serving as auxiliary evaluation tools.  Exploratory factor analysis (EFA): Following the statistical methodology of Kim et al. [13], EFA was performed on symptoms to identify symptom clusters. Prior to factor analysis, the Kaiser-Meyer-Olkin (KMO) measure was used to test partial correlations among variables, with values ranging from 0 to 1; values closer to 1 indicate stronger inter-variable correlations. Bartlett's test of sphericity was employed to verify inter-variable correlations, with P < 0.05 indicating suitability for EFA. Principal component analysis with varimax orthogonal rotation was utilized to extract common factors (symptom clusters). Symptom clusters were subsequently labeled based on constituent symptom characteristics, relevant literature, and clinical expertise.  Network analysis: Network models were constructed using R software, with visualization performed using the qgraph and bootnet packages. The symptom network comprises nodes and edges, where nodes represent individual symptoms and edges (connecting lines between nodes) represent correlations between symptoms; thicker lines indicate stronger associations. Central indices were identified using the networktools package in R. Network centrality indices primarily include three measures: strength centrality, closeness centrality, and betweenness centrality. Strength centrality represents the sum of edge weights connecting a node to all directly linked nodes, reflecting the node's position within the network. Betweenness centrality indicates the number of shortest paths passing through a node, measuring its importance in connecting other nodes. Closeness centrality is the reciprocal of the average shortest path length between nodes, reflecting a node's proximity to other nodes [9].  In symptom networks, strength centrality is the most stable index [14]. Therefore, this study selected strength as the primary criterion for identifying core symptoms, facilitating identification of key intervention targets for healthcare professionals [15]. The bootnet package was utilized to assess centrality index stability following sample reduction, with calculation of the correlation stability coefficient (CS-coefficient). The CS-coefficient should be at least 0.25, preferably exceeding 0.50 [16]. Statistical significance was set at P < 0.05. |
|  |  | (*b*) Describe any methods used to examine subgroups and interactions |  | N/A |
|  |  | (*c*) Explain how missing data were addressed |  | N/A |
|  |  | (*d*) *Cohort study*—If applicable, explain how loss to follow-up was addressed  *Case-control study*—If applicable, explain how matching of cases and controls was addressed  *Cross-sectional study*—If applicable, describe analytical methods taking account of sampling strategy |  | N/A |
|  |  | (*e*) Describe any sensitivity analyses |  | N/A |
| Results | | | | |
| Participants | 13* | (a) Report numbers of individuals at each stage of study—eg numbers potentially eligible, examined for eligibility, confirmed eligible, included in the study, completing follow-up, and analysed |  | N/A |
|  |  | (b) Give reasons for non-participation at each stage |  | N/A |
|  |  | (c) Consider use of a flow diagram |  | N/A |
| Descriptive data | 14* | (a) Give characteristics of study participants (eg demographic, clinical, social) and information on exposures and potential confounders |  | N/A |
|  |  | (b) Indicate number of participants with missing data for each variable of interest |  | N/A |
|  |  | (c) *Cohort study*—Summarise follow-up time (eg, average and total amount) |  | N/A |
| Outcome data | 15* | *Cohort study*—Report numbers of outcome events or summary measures over time |  | N/A |
|  |  | *Case-control study—*Report numbers in each exposure category, or summary measures of exposure |  | N/A |
|  |  | *Cross-sectional study—*Report numbers of outcome events or summary measures |  | N/A |
| Main results | 16 | (*a*) Give unadjusted estimates and, if applicable, confounder-adjusted estimates and their precision (eg, 95% confidence interval). Make clear which confounders were adjusted for and why they were included | 7 | Results revealed that the five most prevalent symptoms in MHD patients were difficulty falling asleep (53.19%), fatigue or tiredness (49.40%), frequent awakening (47.21%), itching (39.44%), and muscle cramps (37.05%). The five symptoms with the highest severity scores were fatigue or tiredness (1.55±1.68), difficulty falling asleep (1.16±1.26), dry mouth (1.03±1.46), muscle cramps (0.96±1.47), and itching (0.89±1.38). Detailed results are presented in Supplementary Table 1. |
|  |  | (*b*) Report category boundaries when continuous variables were categorized |  | N/A |
|  |  | (*c*) If relevant, consider translating estimates of relative risk into absolute risk for a meaningful time period | 7,8 | According to previous research, symptoms with low prevalence cannot reflect the key characteristics of symptom clusters [17]. A total of 21 symptoms were extracted for symptom cluster analysis: constipation, nausea, vomiting, decreased appetite, muscle cramps, lower extremity swelling, shortness of breath, fatigue or tiredness, cough, dry mouth, bone or joint pain, muscle pain, dry skin, itching, worrying, nervousness, difficulty falling asleep, easy awakening, irritability, feeling sad, and anxiety. The Kaiser-Meyer-Olkin (KMO) measure yielded a value of 0.77, and Bartlett's test of sphericity showed χ²=4555.76 (df=210), P<0.001, indicating correlations among symptoms and suitability for factor analysis. |

Continued on next page

| Other analyses | 17 | Report other analyses done—eg analyses of subgroups and interactions, and sensitivity analyses |  | N/A |
| --- | --- | --- | --- | --- |
| Discussion | | | | |
| Key results | 18 | Summarise key results with reference to study objectives | 9,10 | This study enrolled 502 MHD patients, with individuals aged over 50 years accounting for a substantial proportion. The mean age was 52.03±12.16 years, indicating that the MHD population predominantly consisted of middle-aged and elderly individuals. Patients with a dialysis vintage of less than five years constituted the majority (75.2%), with a median dialysis vintage of 42 months, consistent with findings from the national hemodialysis case information system regarding patient demographics [18]. The longest dialysis vintage exceeded 22 years, while the shortest was exactly 3 months. |
| Limitations | 19 | Discuss limitations of the study, taking into account sources of potential bias or imprecision. Discuss both direction and magnitude of any potential bias |  | N/A |
| Interpretation | 20 | Give a cautious overall interpretation of results considering objectives, limitations, multiplicity of analyses, results from similar studies, and other relevant evidence |  | N/A |
| Generalisability | 21 | Discuss the generalisability (external validity) of the study results | 16 | Fatigue and muscle cramps function as pivotal symptom network nodes in maintenance hemodialysis patients. Clinicians should prioritize systematic screening and evidence-based management of these core symptoms—including ultrafiltration optimization, dialysate adjustment, and psychological support—to achieve cascading improvements across interconnected symptom clusters. Network-informed multidisciplinary interventions targeting high-centrality symptoms may effectively reduce overall symptom burden and enhance quality of life.  long-term follow-up for all patients limited our assessment of the treatment's long-term outcomes. |
| Other information | |  | | |
| Funding | 22 | Give the source of funding and the role of the funders for the present study and, if applicable, for the original study on which the present article is based |  | N/A |

*Give information separately for cases and controls in case-control studies and, if applicable, for exposed and unexposed groups in cohort and cross-sectional studies.

**Note:** An Explanation and Elaboration article discusses each checklist item and gives methodological background and published examples of transparent reporting. The STROBE checklist is best used in conjunction with this article (freely available on the Web sites of PLoS Medicine at http://www.plosmedicine.org/, Annals of Internal Medicine at http://www.annals.org/, and Epidemiology at http://www.epidem.com/). Information on the STROBE Initiative is available at www.strobe-statement.org.
