# Supplementary material for: Symptom network analysis in maintenance hemodialysis patients: a multicenter study
Source: PeerJ. 2026 Sep 23;14:e21741. doi: 10.7717/peerj.21741 (PMC13615734; doi:10.7717/peerj.21741)
Supplement: Supplementary file 3 [file peerj-14-21741-s006.docx]

Dear Editor,

Thank you for your attention to the copyright issue regarding the Dialysis Symptom Index (DSI) used in our manuscript. We would like to clarify that the DSI is a publicly available research instrument originally developed and published by Weisbord et al. (2004) in the Journal of Pain and Symptom Management. Since its publication, it has been widely adopted in academic research without the requirement of formal copyright permission, as it is not a proprietary or commercially licensed tool. In accordance with standard academic practice, we have properly cited the original development paper throughout our manuscript: Weisbord SD, Fried LF, Arnold RM, et al. Development of a symptom assessment instrument for chronic hemodialysis patients: the Dialysis Symptom Index. J Pain Symptom Manage. 2004;27(3):226-240. The use of the DSI in our study is consistent with its intended purpose as a non-commercial scientific instrument, and we believe that proper citation constitutes sufficient acknowledgment under academic fair use principles. However, if the journal requires additional documentation, we are happy to contact the original authors to obtain a formal written confirmation of permission for academic use. We hope this clarification is satisfactory. Please let us know if you require any further information.

Sincerely,

Huifen Zhao, MD
